# Association between albuminuria and prevalent diabetic retinopathy in type 2 diabetes: a cross-sectional study with exploratory analysis by carotid plaque status

**DOI:** 10.3389/fendo.2026.1866206

**Published:** 2026-07-15

**Authors:** Nan Li, Huihui Wu, Miaomiao Xu, Xiuming Zhu, Yiming Li, Jie Wen, Yuehua Zhao, Jiong Wang, Chi Liu

**Affiliations:** 1 Department of Geriatrics Center and National Clinical Research Center for Aging and Medicine, Jing’an District Centre Hospital of Shanghai, Fudan University, Shanghai, China; 2 Department of Endocrinology and Metabolism, Jing’an District Center Hospital of Shanghai, Fudan University, Shanghai, China; 3 Department of Hand and Upper Limb Surgery, Jing’an District Centre Hospital of Shanghai, Fudan University, Shanghai, China; 4 Department of Geriatric Respiratory and Critical Care, The First Affiliated Hospital of Anhui Medical University, Hefei, Anhui, China; 5 Department of Endocrinology and Metabolism, Huashan Hospital of Fudan University, Shanghai, China; 6 Department of Rehabilitation Medicine, Jing’an District Central Hospital of Shanghai, Fudan University, Shanghai, China

**Keywords:** albuminuria, carotid plaque, diabetic retinopathy, endothelial dysfunction, type 2 diabetes

## Abstract

**Background and aim:**

Diabetic retinopathy (DR) and diabetic kidney disease share common microvascular mechanisms, whereas carotid plaque reflects systemic atherosclerotic burden in type 2 diabetes (T2D). We examined the association between urinary albumin-to-creatinine ratio (ACR) and prevalent diabetic retinopathy (DR) in patients with type 2 diabetes and conducted exploratory analyses to assess whether this association differed according to carotid plaque status.

**Methods:**

This cross-sectional study included 502 patients with T2D. DR was assessed using fundus photography, and carotid plaque was evaluated by ultrasonography. Because of its skewed distribution, ACR was natural log-transformed as lnACR. Multivariable logistic regression was used to examine the association between lnACR and DR. Subgroup and interaction analyses were performed according to carotid plaque status.

**Results:**

Among 502 participants, 95 (18.9%) had DR. In the extended logistic regression model adjusted for selected clinical covariates, lnACR was associated with prevalent DR (adjusted OR = 1.291, 95% CI: 1.045–1.595, *P* = 0.018). In a modified Poisson sensitivity analysis using the same covariates, the association was directionally similar but attenuated and did not reach statistical significance (adjusted PR = 1.160, 95% CI: 0.975–1.380, *P* = 0.094). The point estimate for the lnACR–DR association was numerically larger among participants with carotid plaque than among those without plaque, but the multiplicative interaction was not statistically significant (P for interaction = 0.220).

**Conclusions:**

Higher lnACR was associated with prevalent DR in adjusted logistic regression, whereas the corresponding modified Poisson sensitivity analysis showed an attenuated, non-significant association. Carotid plaque-related findings were exploratory, and no statistically significant interaction was observed. Because of the cross-sectional complete-case design, these findings should not be interpreted as evidence of incident DR risk, prediction, or support for changes in ophthalmic screening practice.

## 1 Introduction

Diabetic retinopathy (DR) remains a leading cause of visual impairment and blindness in working-age adults worldwide, with its prevalence projected to rise sharply by 2045 (1). Tight glycemic and blood pressure control are cornerstones of DR prevention, yet a considerable residual risk persists even in well-managed patients, indicating that DR pathogenesis extends beyond hyperglycemia and involves widespread systemic vascular impairment (2, 3). Identifying readily available biomarkers that reflect cumulative systemic vascular damage is critical for comprehensive risk assessment and management of DR (4).

The renal–retinal axis has emerged as a key paradigm in diabetic complications (5). The kidney and retina are both susceptible microvascular organs with similar endothelial barrier structures and hemodynamic characteristics; microvascular injury in one organ often indicates parallel dysfunction in the other (6, 7). Albuminuria, measured by the urinary albumin-to-creatinine ratio (ACR), is a clinically accessible marker of renal microvascular injury and may also reflect broader vascular injury or endothelial-related processes; however, endothelial function was not directly measured in the present study (8, 9). Multiple studies have linked elevated ACR to increased DR risk, but the magnitude of association varies across populations, and the impact of concomitant macrovascular disease remains unclear (10, 11).

In addition to microangiopathy, macrovascular atherosclerosis, represented by carotid plaque, is highly prevalent in T2D and has been associated with DR (12, 13). This supports the common soil hypothesis that diabetic micro- and macrovascular complications share common pathological mechanisms, including chronic inflammation, oxidative stress, and endothelial injury (14, 15). However, the interrelationship among carotid plaque, albuminuria, and DR has not been fully elucidated. To date, most studies have examined the independent associations of albuminuria or carotid plaque with DR separately; few have explored their joint association with prevalent DR, and few studies have examined whether the association between albuminuria and prevalent DR differs according to carotid plaque status, particularly in Southern Han Chinese patients. It remains unknown whether the association between albuminuria and DR varies across individuals with different carotid plaque presentation. Therefore, the primary objective of this cross-sectional study was to examine the association between ACR, expressed as natural log-transformed ACR, and prevalent DR in patients with T2D. As secondary exploratory objectives, we assessed whether this association differed according to carotid plaque status and evaluated the joint distribution of elevated ACR and carotid plaque in relation to DR. Because of the cross-sectional design, analyses involving carotid plaque status and interaction were considered exploratory and hypothesis-generating, and no causal, temporal, or predictive inference was made.

Restricting the cohort to Southern Han Chinese patients from the Shanghai metropolitan area was intended primarily to reduce population heterogeneity related to ancestry, regional clinical practice, and environmental context. Genetic variants, dietary patterns, salt sensitivity, and endothelial function were not directly measured; therefore, this restriction should be interpreted as a design feature to improve internal consistency rather than evidence that these mechanisms were controlled (16, 17).

## 2 Materials and methods

### 2.1 Study design and population

**Figure 1:**
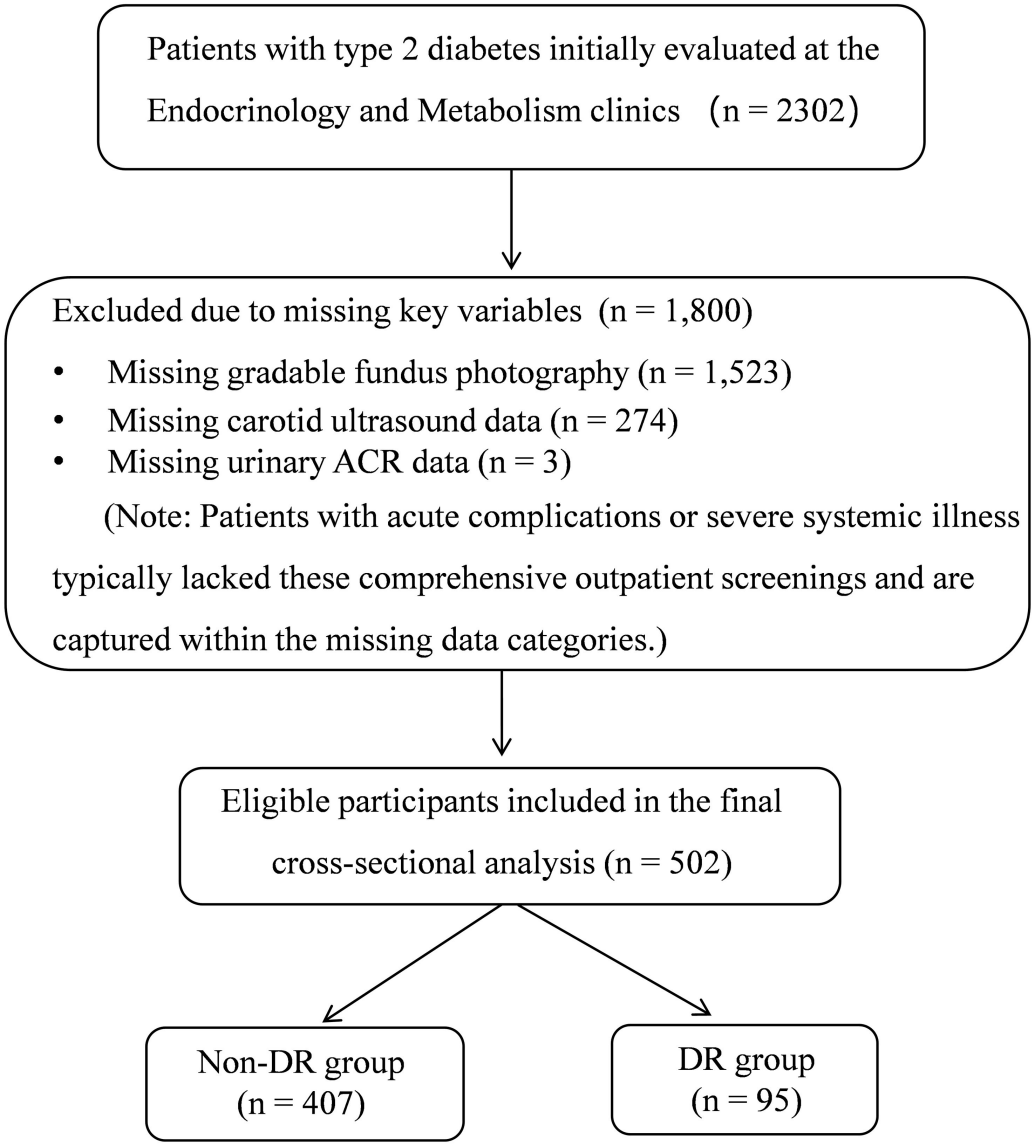
Participant selection and exclusion flow diagram. Flow diagram showing participant identification, exclusion, and final inclusion in the analytic cohort. Of 2,302 patients with type 2 diabetes initially evaluated at the Endocrinology and Metabolism clinics, 1,800 were excluded after application of clinical eligibility criteria or due to missing key study variables. A total of 502 participants were included in the final cross-sectional analysis, including 407 in the non-DR group and 95 in the DR group.

This cross-sectional complete-case study included patients with type 2 diabetes (T2D) of Southern Han Chinese ancestry residing in the Shanghai metropolitan area. The ancestry restriction was used to reduce population heterogeneity. Southern Han Chinese ancestry was defined based on self-reported ethnicity and verified through institutional medical records confirming that the patients’ parents and grandparents were of native Han descent from southern provinces of China. This cohort represents an opportunistic complete-case outpatient screening cohort recruited from the Endocrinology and Metabolism outpatient clinics at Huashan Hospital, Fudan University, between January 2014 and December 2014. Inclusion criteria were as follows: (1) age between 18 and 85 years; (2) outpatients with a confirmed diagnosis of type 2 diabetes; and (3) simultaneous and complete availability of gradable fundus photography, fasting blood chemistry, urinary ACR, and carotid ultrasonography during a single routine visit. The study protocol was approved by the Ethics Committee of Huashan Hospital, Shanghai, China (Approval No. 2014-005), and written informed consent was obtained from all participants. Diabetes was defined as a self-reported history of physician-diagnosed T2D or according to the 1999 WHO criteria as follows: fasting blood glucose ≥7.0 mmol/L, 2-h blood glucose ≥11.1 mmol/L during an oral glucose tolerance test, or random blood glucose ≥11.1 mmol/L. Exclusion criteria were: (1) type 1 diabetes or other specific types of diabetes; (2) history of non-diabetic renal disease; (3) acute diabetic metabolic complications (including diabetic ketoacidosis or hyperosmolar hyperglycemic state), acute urinary tract or systemic infections, and acute kidney injury; (4) severe cardiovascular events, such as myocardial infarction or stroke, within the past 6 months; and (5) missing data on key variables, including ACR, fundus photography, or carotid ultrasound. A total of 502 eligible participants were included in the final analysis. The participant selection process is summarized in **Figure 1**.

During the recruitment period, 2,302 outpatients with T2D were initially screened. Among them, 1,800 were excluded from the final analysis because concurrent key assessments required for this study were unavailable: 1,523 lacked gradable fundus photography, 274 lacked carotid ultrasound data, and 3 lacked urinary ACR data. We could not separately enumerate patients excluded because of acute metabolic complications, acute infection, acute kidney injury, or recent severe cardiovascular events from the historical database. Such patients generally did not complete routine outpatient renal–retinal–carotid screening and were therefore indirectly captured within the missing key-variable categories. A total of 502 participants were included in the final complete-case analysis. The participant selection process is shown in **Figure 1**.

Because complete concurrent testing was required, the analytic cohort should be interpreted as an opportunistic complete-case outpatient screening cohort rather than a consecutive sample of all outpatients with T2D. This selection process may enrich the sample for patients who were older, had longer diabetes duration, or had greater clinical suspicion of vascular complications. Consistent with this possibility, the attrition analysis showed that included participants were older and had longer diabetes duration than excluded individuals, whereas sex distribution, BMI, and HbA1c were broadly similar (**Supplementary Table 2**).

### 2.2 Clinical data collection and laboratory measurements

Demographic information (age, sex, duration of diabetes, smoking, and drinking status) and medical history were collected using a standardized questionnaire. Anthropometric measurements, including height, weight, and systolic/diastolic blood pressure (SBP and DBP), were obtained by trained nurses following standard protocols. Body mass index (BMI) was calculated as weight in kilograms divided by the square of height in meters. Hypertension was defined as a sitting systolic blood pressure (SBP) ≥140 mmHg, a diastolic blood pressure (DBP) ≥90 mmHg, a verified prior physician-documented diagnosis of hypertension, or current use of antihypertensive medication.

Peripheral venous blood samples were collected in the fasting state and 2 h postprandially. The blood was centrifuged at 3,000 rpm for 10 min for plasma separation and immediately used for biomarker assays. Fasting plasma glucose (FPG) and 2-h postprandial glucose (PPG) were quantified using the glucose oxidase-peroxidase procedure. HbA1c was measured by high-performance liquid chromatography using an automated analyzer (HLC-723G7, Tosoh Corporation, Tokyo, Japan). Serum total cholesterol (TC), triglycerides (TG), high-density lipoprotein cholesterol (HDL-C), blood urea nitrogen (BUN), uric acid (UA), and alanine aminotransferase (ALT) levels were measured via enzymatic methods using an autoanalyzer (Hitachi 7600-020, Tokyo, Japan). Low-density lipoprotein cholesterol (LDL-C) levels were calculated using the Friedewald formula; participants with severe hypertriglyceridemia (TG ≥4.5 mmol/L) were excluded from this specific calculation. The estimated glomerular filtration rate (eGFR) was calculated using the Modification of Diet in Renal Disease (MDRD) equation. The intra-assay and inter-assay coefficients of variation at our central laboratory for all analyses were consistently between 1% and 3%.

### 2.3 Assessment of urinary albumin-to-creatinine ratio

First-morning spot urine samples were collected from all participants. Urinary albumin and creatinine concentrations were measured using immunoturbidimetric and enzymatic methods, respectively, and albumin-to-creatinine ratio (ACR) was calculated as urinary albumin divided by urinary creatinine. ACR was measured once at baseline. Participants with clinically evident urinary tract infection, fever, acute kidney injury, acute systemic illness, or other acute conditions at the time of urine collection were excluded when such information was available. Information on vigorous exercise immediately before urine collection was not available in this retrospective dataset. Repeated ACR measurements were not available; therefore, potential misclassification due to biological variability in albuminuria cannot be excluded. Because ACR was measured only once, elevated ACR in this study should be interpreted as a single baseline albuminuria marker rather than persistent albuminuria or established diabetic kidney disease.

In addition to modeling ACR as a natural log-transformed continuous variable, we conducted clinically interpretable categorical analyses using elevated ACR, defined as ≥3.4 mg/mmol, approximately equivalent to ≥30 mg/g. A single, unified threshold of 3.4 mg/mmol was applied for all participants in accordance with the KDIGO clinical practice guidelines for generalized microvascular risk assessment, rather than implementing sex-specific cutoffs.

### 2.4 Assessment of diabetic retinopathy (outcome)

Both eyes of each participant were photographed using a 45° 6.3-megapixel digital non-mydriatic fundus camera (Canon CR6-45NM, Lake Success, NY, USA). Images were repeated when necessary to ensure adequate quality. Images were considered ungradable when the retinal vessels or key retinal landmarks could not be adequately assessed. Participants with ungradable images in both eyes were excluded from the primary analysis. When both eyes were gradable, DR severity was defined according to the eye with the more severe lesion.

Because this study used a retrospective clinical database initiated in 2014, fundus photography was performed as part of routine outpatient care rather than a predefined multi-field ETDRS research protocol. Typically, central macula- and optic disc-centered non-mydriatic images were obtained, and pharmacological dilation was performed only when clinically required. Images were considered ungradable when retinal vessels or key retinal landmarks could not be adequately assessed, and participants with ungradable images in both eyes were excluded. This routine imaging approach may have under-ascertained peripheral retinal lesions compared with standardized ETDRS 7-field imaging. For 24 DR cases with ambiguous historical severity staging, two experienced clinicians retrospectively reviewed the original clinical fundus descriptions. Formal documentation of masking to ACR and carotid plaque status was not archived; disagreements, if any, were resolved by consensus. Formal inter-grader agreement statistics before adjudication were not systematically available. All 95 DR cases were retained for the severity trend analysis after adjudication.

### 2.5 Assessment of carotid atherosclerosis

Carotid ultrasonography was performed in the routine outpatient setting by a single experienced vascular sonographer who was blinded to participants’ clinical, renal, and ophthalmic status. Bilateral common carotid arteries, carotid bulbs, and accessible internal carotid artery segments were examined longitudinally and transversely using a high-resolution B-mode ultrasound system (Affiniti 70, Philips, Amsterdam, Netherlands) with a broadband linear-array transducer operating within an approximately 5–12-MHz range. Carotid plaque was defined according to the Mannheim consensus as a focal structure protruding into the lumen by at least 0.5 mm, or representing an increase of ≥50% relative to the adjacent IMT, or exhibiting an absolute localized thickness >1.5 mm. Plaque burden was quantified as the total number of distinct plaques across scanned bilateral segments (18). However, granular segment-specific plaque counts and formal reproducibility data for plaque counting were not available in this retrospective cohort.

### 2.6 Statistical analysis

Statistical analyses were performed using SPSS version 27.0 (IBM Corp., Armonk, NY, USA). The distribution of continuous variables was assessed primarily through the visual inspection of histograms, complemented by the Kolmogorov–Smirnov test, to avoid overreliance on sample-size-sensitive formal testing. Continuous variables are presented as mean ± standard deviation (SD) for approximately normally distributed data or median and interquartile range (IQR) for skewed data. Categorical variables are presented as frequencies and percentages. Between-group differences were assessed using the independent-samples Student’s t-test, Mann–Whitney U test, or chi-square test, as appropriate.

To evaluate the association between albuminuria and DR, multivariable logistic regression models were constructed with DR as the dependent variable and lnACR as the main independent variable. Covariates were selected *a priori* based on clinical relevance and established risk factors for DR. Three models were fitted. Model 1 was an unadjusted model evaluating the unadjusted association between lnACR and DR. Model 2 adjusted for age and sex. Model 3 adjusted for selected clinical covariates, including age, sex, duration of diabetes, HbA1c, systolic blood pressure, BMI, eGFR, smoking history, and LDL-C. This model was intended to reduce confounding by major measured demographic and cardiometabolic factors; however, important unmeasured factors, including medication use and long-term glycemic history, were unavailable. Multicollinearity was assessed using the variance inflation factor (VIF), and a VIF <5 was considered acceptable. The potential descriptive trend was assessed using trend analysis across ACR quartiles. Prior to model construction, the assumption of linearity in the logit for the continuous variable (lnACR) was verified by testing its quadratic term; the non-linear term was not statistically significant (*P* = 0.226), indicating the linearity assumption was satisfied.

To descriptively examine whether the lnACR–DR association differed across carotid plaque strata, exploratory stratified analyses were performed according to carotid plaque status and other clinically relevant variables. Multiplicative interaction was assessed by adding a cross-product term between lnACR and the stratifying variable to the multivariable logistic regression model. Because subgroup analyses were exploratory, results were interpreted according to both effect size and interaction *P* values. Because the prevalence of DR was 18.9%, odds ratios may overestimate prevalence ratios. Therefore, a *post-hoc* modified Poisson regression model with robust variance was performed using the same covariates as model 3 to estimate prevalence ratios as a sensitivity analysis.

Exploratory additive interaction analyses were also conducted by categorizing participants according to the combined presence of carotid plaque and elevated ACR. Elevated ACR was defined as ≥3.4 mg/mmol, equivalent to ≥30 mg/g. The relative excess risk due to interaction (RERI) and attributable proportion due to interaction (AP) were estimated. Because these measures were derived from odds ratios in a cross-sectional study with a non-rare outcome, they were treated as exploratory OR-based approximations and were not interpreted as definitive measures of risk or prevalence additivity.

Trend analyses were performed to evaluate changes in DR prevalence across ACR quartiles and changes in lnACR across DR severity categories. The linear-by-linear association test and one-way ANOVA with polynomial contrasts were used as appropriate. All tests were two-sided, and *P* < 0.05 was considered statistically significant. No adjustment for multiple comparisons was applied; subgroup, interaction, trend, and sensitivity analyses were interpreted conservatively as exploratory.

Missing data were handled using complete-case analysis because urinary ACR, fundus photography, and carotid ultrasound were essential variables for the primary and exploratory analyses. Participants missing any of these key variables were excluded from the final analytic sample. Multiple imputation was not performed for the primary analysis because participants missing the key exposure, outcome, or carotid ultrasound data could not contribute to the planned analyses.

## 3 Results

### 3.1 Baseline characteristics

The participant selection process is shown in **Figure 1**. After application of the predefined eligibility criteria, 502 participants were included in the final analysis, including 407 participants without DR, and 95 participants with DR. Baseline characteristics of the study participants stratified by DR status are presented in **Table 1**. Compared with participants without DR, participants with DR had a longer duration of diabetes (8.69 ± 6.55 vs. 6.95 ± 6.08 years, *P* = 0.015), higher fasting plasma glucose (10.06 ± 3.88 vs. 8.32 ± 2.81 mmol/L, *P* < 0.001), and higher HbA1c levels (8.04 ± 1.88% vs. 6.98 ± 1.45%, *P* < 0.001). The DR group also had higher lnACR (1.56 ± 1.28 vs. 1.21 ± 1.10, *P* = 0.008). Systolic blood pressure was numerically higher in participants with DR, but the difference did not reach statistical significance (141.08 ± 18.95 vs. 136.97 ± 20.75 mmHg, *P* = 0.078). The proportion of elevated ACR (≥3.4 mg/mmol) did not differ significantly between groups (49.5% vs. 43.2%, *P* = 0.271). Smoking history was less frequent in the DR group (3.2% vs. 10.1%, *P* = 0.031). No statistically significant differences were observed in age, sex distribution, BMI, lipid profiles, uric acid, diastolic blood pressure, eGFR, or carotid plaque prevalence (*P* > 0.05 for all).

**Table 1:** Baseline characteristics of the study population stratified by diabetic retinopathy status.

Variables	Non-DR (n=407)	DR (n=95)	*P* value
Demographics			
Age (years)	64.62 ± 10.73	64.61 ± 10.34	0.993
Male sex, n (%)	152 (37.3%)	38 (40.0%)	0.631
Smoking history, n (%)	41 (10.1%)	3 (3.2%)	0.031
Drinking history, n (%)	24 (5.9%)	2 (2.1%)	0.131
Duration of diabetes (years)	6.95 ± 6.08	8.69 ± 6.55	0.015
Anthropometry			
BMI (kg/m²)	24.81 ± 3.36	24.83 ± 3.26	0.968
SBP (mmHg)	136.97 ± 20.75	141.08 ± 18.95	0.078
DBP (mmHg)	81.21 ± 10.99	81.86 ± 13.31	0.659
Metabolic profile			
HbA1c (%)	6.98 ± 1.45	8.04 ± 1.88	< 0.001
ACR (mg/mmol)	2.72 (1.37–7.06)	3.36 (1.75–8.91)	0.063
Elevated ACR (≥3.4 mg/mmol), n (%)	176 (43.2%)	47 (49.5%)	0.271
TG (mmol/L)	1.97 ± 1.37	2.10 ± 1.50	0.43
TC (mmol/L)	5.36 ± 1.10	5.40 ± 1.20	0.78
HDL-C (mmol/L)	1.28 ± 0.34	1.28 ± 0.39	0.948
LDL-C (mmol/L)	3.06 ± 0.82	3.09 ± 0.91	0.766
UA (µmol/L)	291.8 ± 75.9	285.2 ± 76.6	0.444
eGFR (MDRD)	98.15 ± 21.81	94.90 ± 25.05	0.205
FPG (mmol/L)	8.32 ± 2.81	10.06 ± 3.88	< 0.001
Carotid plaque, n (%)	143 (35.1%)	34 (35.8%)	0.904

Data are presented as mean ± standard deviation for approximately normally distributed continuous variables, median and interquartile range for skewed continuous variables, or number and percentage for categorical variables. *P* values were calculated using the independent-samples Student’s t-test, Mann–Whitney U test, or chi-square test, as appropriate. ACR was recorded and presented in mg/mmol. Elevated ACR was defined as ≥3.4 mg/mmol, approximately equivalent to ≥30 mg/g.Abbreviations: ACR, albumin-to-creatinine ratio; BMI, body mass index; DBP, diastolic blood pressure; DR, diabetic retinopathy; eGFR, estimated glomerular filtration rate; FPG, fasting plasma glucose; HbA1c, glycated hemoglobin; HDL-C, high-density lipoprotein cholesterol; LDL-C, low-density lipoprotein cholesterol; SBP, systolic blood pressure; TC, total cholesterol; TG, triglycerides; UA, uric acid.

An attrition analysis comparing included and excluded individuals is presented in **Supplementary Table 2**. Compared with excluded individuals, included participants were older (64.6 ± 10.6 vs. 61.7 ± 9.5 years, *P* < 0.001) and had longer diabetes duration (7.3 ± 6.2 vs. 3.6 ± 6.5 years, *P* < 0.001), whereas sex distribution, BMI, and HbA1c were broadly similar.

### 3.2 Trends across albuminuria and DR severity

**Figure 2:**
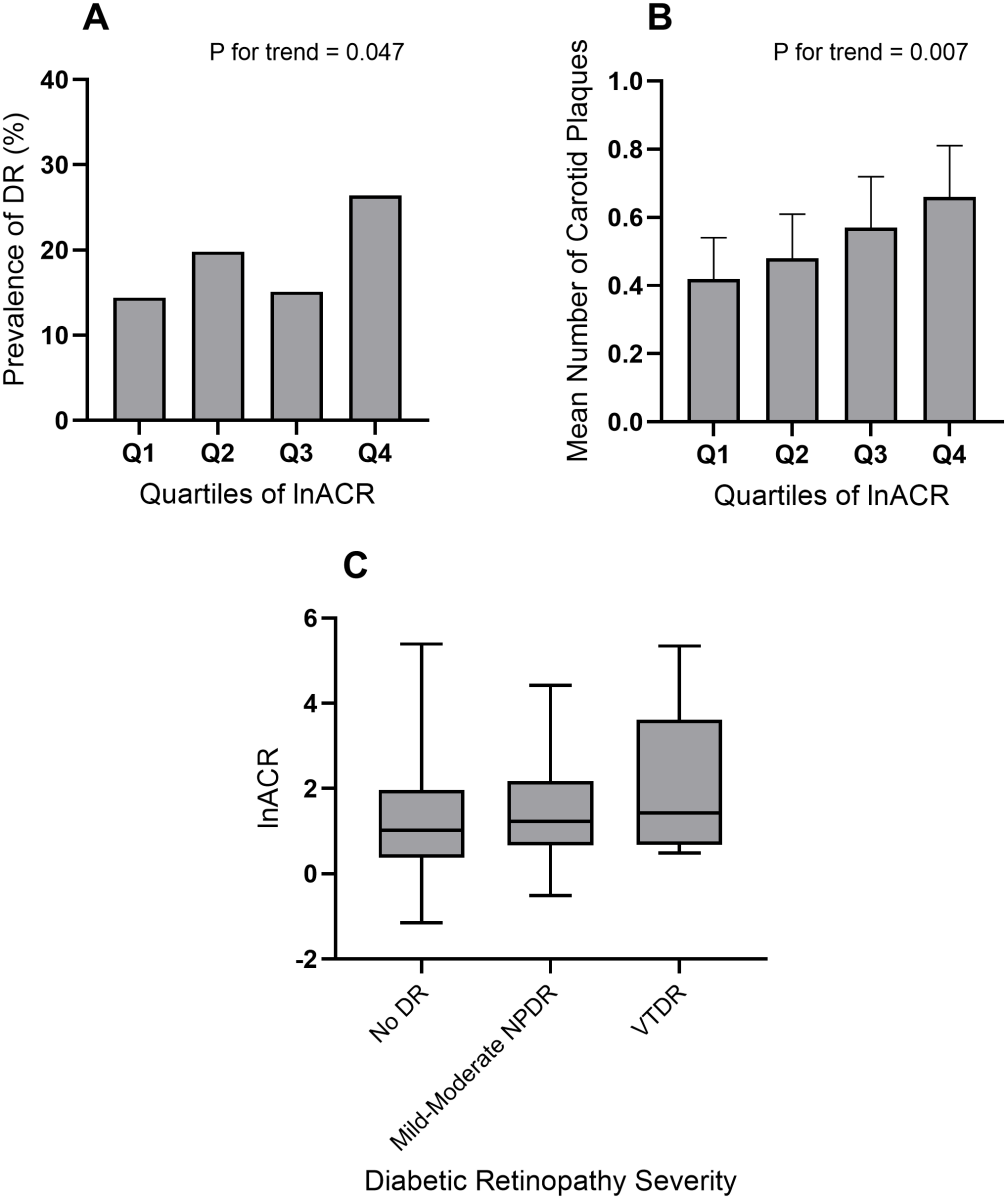
Trends across albuminuria, carotid plaque burden, and diabetic retinopathy severity. **(A)** Prevalence of diabetic retinopathy across quartiles of urinary albumin-to-creatinine ratio (ACR). DR prevalence was highest in the upper quartile, and the overall trend test was statistically significant (P for trend = 0.047), although the pattern was not strictly monotonic across all quartiles. ACR quartile cut points were: Q1 ≤ 1.42 mg/mmol; Q2, 1.43–2.74 mg/mmol; Q3, 2.75–7.28 mg/mmol; and Q4, > 7.28 mg/mmol. **(B)** Mean number of carotid plaques across ACR quartiles (P for trend = 0.007). **(C)** Natural log-transformed ACR (lnACR) across DR severity categories (P for trend = 0.005). All 95 DR cases were included in the severity trend analysis after retrospective adjudication of 24 cases with initially ambiguous staging. Data were derived from 502 patients with type 2 diabetes. Error bars in panels A and B indicate 95% confidence intervals. In panel C, box plots display the median, interquartile range, and values within 1.5 times the interquartile range, with outliers shown as individual points. P values for trend were calculated using the linear-by-linear association test for categorical proportions and one-way ANOVA with polynomial contrasts for continuous variables.

To further explore the descriptive trends among renal microvascular injury, carotid macrovascular atherosclerosis, we analyzed variables across different clinical strata (**Figure 2**). **Figure 2** summarizes exploratory trends across ACR quartiles and DR severity categories. DR prevalence was highest in the upper ACR quartile, and the overall trend test across quartiles was statistically significant (P for trend = 0.047; **Figure 2A**), although the pattern was not strictly monotonic across all quartiles. Mean carotid plaque burden increased across ACR quartiles (P for trend = 0.007; **Figure 2B**), with the mean number of carotid plaques rising from 0.42 (95% CI: 0.29–0.54) in Q1 to 0.48 (95% CI: 0.34–0.61) in Q2, 0.57 (95% CI: 0.43–0.72) in Q3, and 0.66 (95% CI: 0.52–0.81) in Q4. lnACR showed a positive trend across DR severity categories (P for trend = 0.005; **Figure 2C**). All 95 DR cases were included in the DR severity trend analysis after retrospective adjudication of 24 cases with initially ambiguous staging.

### 3.3 Independent association between lnACR and DR

Multivariable logistic regression results are summarized in **Table 2**. In model 1, lnACR was associated with DR in the unadjusted model (OR = 1.285, 95% CI: 1.065–1.550). The estimate was similar after adjustment for age and sex in model 2 (OR = 1.289, 95% CI: 1.067–1.558) and after adjustment for selected clinical covariates in model 3 (adjusted OR = 1.291, 95% CI: 1.045–1.595, P = 0.018). There was no evidence of severe multicollinearity among the covariates in model 3 (VIF range: 1.05 to 1.44). The model 3 estimate corresponds approximately to a 19% higher odds of DR per doubling of ACR.

**Table 2:** Logistic regression and modified Poisson sensitivity analysis for the association between lnACR and diabetic retinopathy.

Variables	Model 1 (basic) estimate (95% CI)	*P* value (M1)	Model 2 (main clinical) estimate (95% CI)	*P* value (M2)	Model 3 (extended sensitivity) estimate (95% CI)	*P* value (M3)
Number of participants (n)	502	–	502	–	502	–
DR events (n)	95	–	95	–	95	–
Primary analysis						
lnACR, logistic OR	1.285 (1.065–1.550)	0.009	1.289 (1.067–1.558)	0.009	1.291 (1.045–1.595)	0.018
Sensitivity analysis						
lnACR, modified Poisson PR	–	–	–	–	1.160 (0.975–1.380)	0.094
Adjusted covariates						
Age (years)	–	–	0.997 (0.976–1.019)	0.794	0.958 (0.925–0.993)	0.020
Male sex	–	–	0.876 (0.552–1.390)	0.575	0.938 (0.553–1.591)	0.812
Duration of diabetes (years)	–	–	–	–	1.032 (0.993–1.073)	0.110
HbA1c (%)	–	–	–	–	1.487 (1.278–1.731)	< 0.001
SBP (mmHg)	–	–	–	–	1.006 (0.993–1.019)	0.352
BMI (kg/m^2^)	–	–	–	–	0.994 (0.923–1.070)	0.874
eGFR (mL/min/1.73m^2^)	–	–	–	–	0.975 (0.957–0.994)	0.010
Smoking history	–	–	–	–	0.246 (0.069–0.874)	0.030
LDL-C (mmol/L)	–	–	–	–	0.939 (0.698–1.262)	0.674

Logistic regression results are presented as odds ratios (ORs) and 95% confidence intervals (CIs). Model 1 was unadjusted. Model 2 was adjusted for age and sex. Model 3 was adjusted for selected clinical covariates, including age, sex, duration of diabetes, HbA1c, systolic blood pressure (SBP), body mass index (BMI), estimated glomerular filtration rate (eGFR), smoking history, and low-density lipoprotein cholesterol (LDL-C). Modified Poisson regression with robust variance was performed using the same covariates as model 3 and is presented as a prevalence ratio (PR) and 95% CI. P values are shown to facilitate comparison across model specifications. ACR, albumin-to-creatinine ratio; BMI, body mass index; CI, confidence interval; DR, diabetic retinopathy; eGFR, estimated glomerular filtration rate; HbA1c, glycated hemoglobin; lnACR, natural log-transformed ACR; LDL-C, low-density lipoprotein cholesterol; OR, odds ratio; PR, prevalence ratio; SBP, systolic blood pressure.

Because the overall prevalence of DR was 18.9%, logistic regression odds ratios may overestimate prevalence ratios. Therefore, we performed a *post-hoc* modified Poisson regression model with robust variance using the same covariates as model 3. In this sensitivity analysis, lnACR showed a directionally similar but smaller estimate that did not reach statistical significance (adjusted PR = 1.160, 95% CI: 0.975–1.380, *P* = 0.094). This suggests that the logistic odds ratio may overstate the magnitude of association, and the main association should therefore be interpreted descriptively.

### 3.4 Exploratory subgroup and interaction analyses

**Figure 3:**
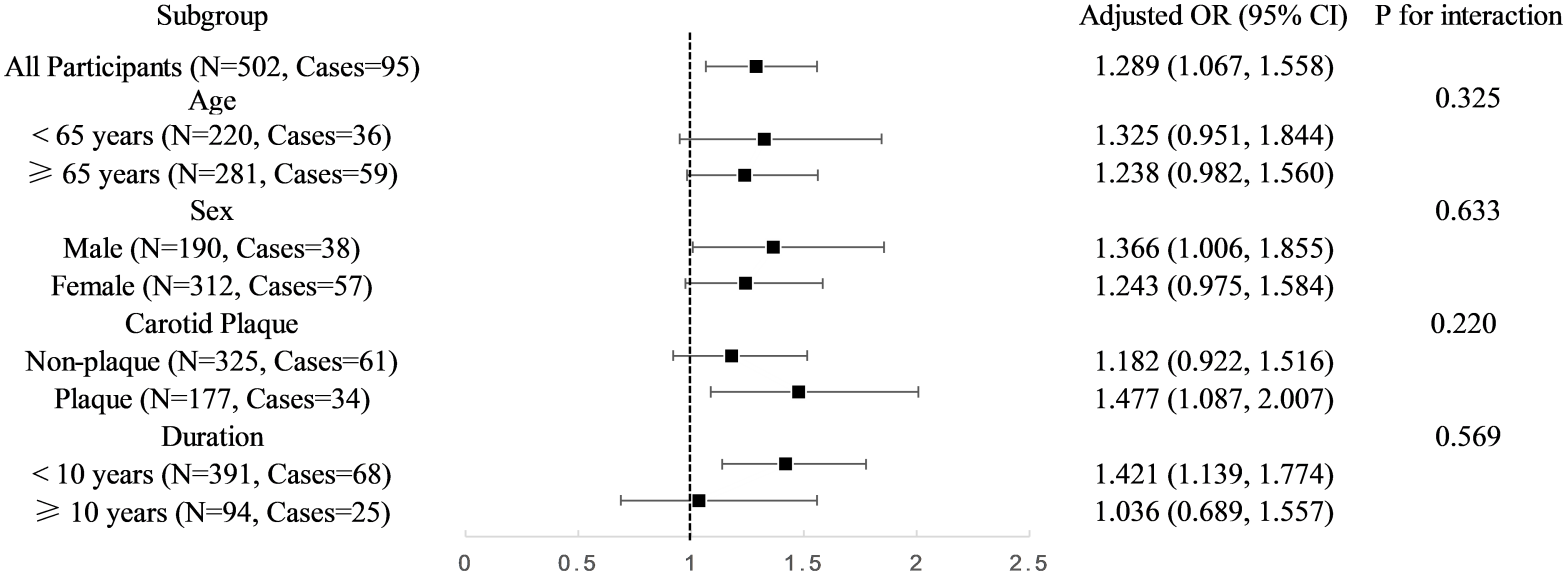
Exploratory subgroup analyses of the association between lnACR and diabetic retinopathy. The forest plot shows adjusted odds ratios and 95% confidence intervals for the association between lnACR and diabetic retinopathy across prespecified clinical subgroups. To preserve statistical power within smaller strata, subgroup analyses were performed using the core clinical model adjusted for age, sex, duration of diabetes, HbA1c, and systolic blood pressure, rather than the extended sensitivity model. The stratification variable was omitted from the corresponding subgroup model. Solid squares indicate point estimates, horizontal lines indicate 95% confidence intervals, and the vertical dashed line at OR = 1.0 represents the null value. P for interaction values were obtained by adding multiplicative cross-product terms to the regression models. These analyses were exploratory and were not adjusted for multiple comparisons. CI, confidence interval; lnACR, natural log-transformed albumin-to-creatinine ratio; OR, odds ratio.

Subgroup analyses are shown in **Figure 3**. These exploratory analyses used the core clinical model adjusted for age, sex, diabetes duration, HbA1c, and systolic blood pressure, rather than the extended model 3, to preserve statistical power within smaller strata. Therefore, subgroup estimates may be more vulnerable to residual confounding. When stratified by carotid plaque status, the lnACR–DR estimate was numerically larger among participants with carotid plaque (adjusted OR = 1.477, 95% CI: 1.087–2.007) than among those without plaque (adjusted OR = 1.182, 95% CI: 0.922–1.516). However, the multiplicative interaction was not statistically significant (P for interaction = 0.220), and these findings should not be interpreted as evidence of confirmed effect modification.

In the joint exposure analysis, participants were categorized according to carotid plaque status and elevated ACR. Compared with participants with neither carotid plaque nor elevated ACR, the adjusted ORs were 0.79 (95% CI: 0.38–1.65) for carotid plaque alone, 0.97 (95% CI: 0.53–1.77) for elevated ACR alone, and 1.23 (95% CI: 0.63–2.39) for both carotid plaque and elevated ACR (**Supplementary Table 1**). The multiplicative interaction was not statistically significant (P = 0.180). The OR-based RERI was 0.467 (95% CI: −0.48 to 1.41), and the AP was 38.0% (95% CI: −31.8% to 107.8%). Because the joint exposure estimate was imprecise and the confidence intervals for interaction metrics crossed the null, these findings are inconclusive and provide no definitive evidence of additive or multiplicative interaction.

## 4 Discussion

In this cross-sectional complete-case outpatient screening cohort of 502 patients with type 2 diabetes, higher lnACR was associated with prevalent DR in adjusted logistic regression. However, the association was attenuated and did not reach statistical significance in the modified Poisson sensitivity analysis. Therefore, the findings support a cautious association between a single baseline albuminuria marker and prevalent DR rather than a robust predictive or causal relationship. In exploratory subgroup analyses, the point estimate for the lnACR–DR association was numerically larger among participants with carotid plaque, but the formal interaction test was not statistically significant.

The association between albuminuria and DR is consistent with the renal–retinal framework in diabetes (6, 19, 20). The kidney and retina are both highly vascularized microvascular organs and are vulnerable to chronic metabolic, hemodynamic, and inflammatory stress. Increased urinary albumin excretion may reflect impaired glomerular permeability and broader endothelial-related vascular injury, which may coexist with microvascular damage in the retina. However, endothelial function was not directly measured in this study, and ACR was assessed only once. Thus, the exposure should not be interpreted as persistent albuminuria or established diabetic kidney disease.

The numerically different stratified estimates by carotid plaque status require conservative interpretation. Although the point estimate was higher among participants with carotid plaque, the interaction test did not reach statistical significance. The observed difference may reflect limited sample size, residual confounding, sparse events in some strata, or shared systemic vascular burden rather than true biological effect modification. Larger longitudinal studies are required before carotid plaque can be considered a clinically useful contextual marker for interpreting the albuminuria–DR association.

The lack of a clear independent relationship between carotid plaque and DR in this cohort should be interpreted in light of recent prospective evidence. In two prospective cohorts from southern China, Chen et al. reported that a 10-year ASCVD risk score predicted incident diabetic nephropathy but did not predict incident DR among patients with T2D. This finding argues against overinterpreting macrovascular atherosclerotic burden as a direct determinant or validated risk-stratification marker for DR. Consistently, our carotid plaque subgroup and joint exposure analyses did not show statistically significant interaction, and carotid plaque should be viewed only as an exploratory contextual vascular marker in the present cross-sectional study.

From a clinical perspective, the present findings support further investigation of renal and vascular markers in longitudinal DR studies, but they do not justify changes to ophthalmic screening intervals or clinical decision-making. This study did not evaluate incident DR, model discrimination, calibration, reclassification, sensitivity, specificity, or decision-curve performance. Future studies should determine whether albuminuria, alone or in combination with carotid vascular markers, provides incremental predictive value beyond established DR risk factors.

Several limitations should be acknowledged. First, the cross-sectional design precludes causal inference and does not allow determination of temporal relationships among carotid plaque, albuminuria, and DR. Second, selection bias may have arisen from non-random complete-case inclusion, as 1,800 of 2,302 initially screened patients did not have concurrent key assessments. The final cohort represents patients who completed renal, retinal, and carotid evaluations in routine outpatient care and should not be generalized to the broader outpatient T2D population. As shown in **Supplementary Table 2**, included participants were older and had longer diabetes duration than excluded individuals. Third, the single-center Southern Han Chinese cohort limits generalizability to other populations. Fourth, residual confounding cannot be excluded because medication use, including renin–angiotensin system blockade, statins, insulin, SGLT2 inhibitors, GLP-1 receptor agonists, and other glucose-lowering therapies, as well as long-term glycemic history, socioeconomic factors, healthcare utilization, and prior ophthalmic screening history were unavailable.

Fifth, ophthalmic and ultrasonographic evaluations reflected routine 2014 outpatient practice rather than standardized research protocols. Routine non-mydriatic fundus photography may have under-ascertained peripheral retinal lesions compared with ETDRS 7-field imaging. Formal inter-grader reliability for DR grading and formal reproducibility data for carotid plaque counting were not systematically archived, so outcome and plaque-burden measurement variability cannot be excluded. Sixth, DME was not systematically assessed, so VTDR in this study did not represent the full standard clinical definition of vision-threatening diabetic eye disease. Seventh, ACR was measured only once, and transient or biological variability may have caused exposure misclassification. Eighth, given the 18.9% prevalence of DR, logistic odds ratios may overestimate prevalence ratios; the modified Poisson sensitivity analysis yielded an attenuated, non-significant estimate. Finally, although DR prevalence was highest in the upper ACR quartile and the overall trend test was statistically significant, the quartile pattern was not strictly monotonic, and no restricted cubic spline analysis was performed to characterize the continuous dose–response shape.

Despite these limitations, this study has several strengths. It simultaneously evaluated urinary albumin excretion, carotid atherosclerosis, and retinal complications in a clinically characterized T2D population. The revised analysis focuses on the adjusted association between lnACR and prevalent DR and presents carotid plaque-related analyses as exploratory. These findings provide descriptive clinical evidence supporting further longitudinal investigation of systemic vascular markers in DR research (21, 22).

## Data Availability

The original contributions presented in the study are included in the article/**Supplementary Material**. Further inquiries can be directed to the corresponding authors.
