# Supplementary material for: Association between albuminuria and prevalent diabetic retinopathy in type 2 diabetes: a cross-sectional study with exploratory analysis by carotid plaque status
Source: Front Endocrinol (Lausanne). 2026 Jul 15;17:1866206. doi: 10.3389/fendo.2026.1866206 (PMC13414848; doi:10.3389/fendo.2026.1866206)
Supplement: Supplementary file 1 [file Table1.docx]

**Table S1. Joint association and exploratory additive interaction of carotid plaque and elevated ACR with diabetic retinopathy**

| **Carotid plaque** | **Elevated ACR (≥3.4 mg/mmol)** | **Total N** | **DR Cases** | **Adjusted OR (95% CI) *** |
| --- | --- | --- | --- | --- |
| No | No | 184 | 34 | 1.00 (Reference) |
| Yes | No | 81 | 11 | 0.79 (0.38–1.65) |
| No | Yes | 141 | 27 | 0.97 (0.53–1.77) |
| Yes | Yes | 98 | 25 | 1.23 (0.63–2.39) |

Measures of Interaction:

- Multiplicative interaction: *P* = 0.180
- Additive interaction: * Relative excess risk due to interaction (RERI) = 0.467 (95% CI: -0.48 to 1.41)

Attributable proportion (AP) = 38.0% (95% CI: -31.8% to 107.8%)

* Adjusted for age, sex, duration of diabetes, HbA1c, and systolic blood pressure.

**Notes:** The point estimates for RERI and AP were positive; however, the confidence intervals included the null, and the multiplicative interaction test was not statistically significant. Because these interaction measures were derived from odds ratios in a cross-sectional study with a non-rare outcome, they should be interpreted as exploratory OR-based approximations rather than definitive evidence of risk or prevalence additivity. Overall, the joint exposure findings were inconclusive and provided no definitive evidence of additive or multiplicative interaction.

**Abbreviations:** ACR, albumin-to-creatinine ratio; AP, attributable proportion; CI, confidence interval; DR, diabetic retinopathy; OR, odds ratio; RERI, relative excess risk due to interaction.

**Table S2. Comparison of baseline characteristics between the included and excluded cohorts**

| **Baseline Characteristics** | **Included Cohort (n=502)** | **Excluded Cohort (n=1,800)** | ***P* value** |
| --- | --- | --- | --- |
| Age (years) | 64.6±10.6 | 61.7±9.5 | < 0.001 |
| Male sex, n (%) | 191 (37.9%) | 617 (34.3%) | 0.137 |
| Duration of diabetes (years) | 7.3±6.2 | 3.6±6.5 | < 0.001 |
| BMI (kg/m^2^) | 24.8±3.3 | 24.7±3.3 | 0.375 |
| HbA1c (%) | 7.2±1.6 | 7.4±1.7 | 0.053 |

**Notes**: Data are presented as mean ± standard deviation (SD) for continuous variables or number and percentage, n (%), for categorical variables. *P* values were calculated using the independent-samples Student’s t-test or the chi-square test, as appropriate. The attrition analysis reveals that while the included and excluded cohorts were well-matched regarding sex distribution, BMI, and glycemic control (HbA1c), the excluded individuals were younger and had a significantly shorter duration of diabetes. The included participants were older and had longer diabetes duration than excluded individuals. This pattern suggests potential selection bias related to complete-case inclusion and may reflect real-world outpatient screening practices; however, the exact reasons for incomplete concurrent testing could not be fully determined from the historical database.

**Abbreviations:** BMI, body mass index; HbA1c, glycated hemoglobin.
